# Ultra‐processed food intake, cognitive function, and dementia risk: A cross‐sectional study of middle‐aged and older Australian adults

**DOI:** 10.1002/dad2.70335

**Published:** 2026-04-23

**Authors:** Barbara R. Cardoso, Euridice Martinez Steele, Barbara Brayner, Xinyi Yuan, Lisa Bransby, Hannah Cummins, Yen Ying Lim, Priscila Machado

**Affiliations:** ^1^ Department of Nutrition Dietetics and Food, Monash University Clayton VIC Australia; ^2^ Victorian Heart Institute Monash University Clayton VIC Australia; ^3^ Department of Nutrition, School of Public Health University of São Paulo São Paulo SP Brazil; ^4^ Center for Epidemiological Studies in Health and Nutrition University of São Paulo São Paulo SP Brazil; ^5^ Institute for Physical Activity and Nutrition (IPAN), School of Exercise and Nutrition Sciences Deakin University Burwood VIC Australia; ^6^ Turner Institute for Brain and Mental Health, School of Psychological Sciences Monash University Clayton VIC Australia

**Keywords:** cognition, dementia risk, diet, healthy diet, Mediterranean diet, nutrition, ultra‐processed foods

## Abstract

**INTRODUCTION:**

Ultra‐processed food (UPF) consumption is linked to over 30 adverse health outcomes, including several risk factors for dementia such as cardiovascular disease, type 2 diabetes, and obesity. We aimed to examine the association of UPF consumption with cognitive performance and dementia risk scores, and whether these associations are independent of overall diet quality.

**METHODS:**

This cross‐sectional analysis assessed 2,192 Australian dementia‐free adults aged 40–70 years. Diet was assessed using a validated food frequency questionnaire and classified according to the Nova system. Cognitive function was measured using the Cogstate Brief Battery, and dementia risk was estimated with the CAIDE tool.

**RESULTS:**

Each 10% increase in UPF intake was associated with lower attention scores (−0.05 points) and higher dementia risk (+0.24 points), independent of Mediterranean diet adherence.

**DISCUSSION:**

Higher UPF consumption is associated with poorer attention and increased modifiable dementia risk, independent of overall diet quality.

## Highlights

- Higher ultra‐processed food (UPF) intake is linked to poorer attention in midlife Australian adults
- UPF consumption is associated with increased modifiable dementia risk scores
- Associations were independent of Mediterranean diet adherence
- No significant link found between UPF intake and memory
- Findings support food processing as a distinct factor in cognitive health

## 1 INTRODUCTION

The global proliferation of ultra‐processed foods (UPFs), defined as industrial formulations composed of refined ingredients and cosmetic additives with minimal whole food content, has raised significant public health concerns.^1^ UPFs now constitute over half of total dietary energy intake in high‐income countries such as the United States and the United Kingdom,^2^ and approximately 42% in Australia.^3^ Similar trends are also emerging across low‐ and middle‐income nations.^4^

While evidence on the role of overall and specific UPFs in the diet continues to emerge,^5^ to date, UPF consumption has been associated with over 30 adverse health outcomes, including cardiovascular diseases, type 2 diabetes, obesity, mental disorders, and mortality.^1^ More recently, concerns have emerged about the potential impact of UPFs on brain health. Several observational studies have reported associations between higher UPF consumption and poorer cognitive performance or accelerated cognitive decline.^6^, ^7^, ^8^ Meta‐analyses have also suggested that UPF intake is associated with elevated risks of cognitive impairment and dementia; however, substantial heterogeneity across studies due to study design, sample size, and definition of UPF limits the quality and generalizability of this evidence.^9^, ^10^ Further, higher UPF consumption often coincides with lower intake of nutrient‐dense foods that support cognitive health. As a result, the unique contribution of UPF beyond overall diet quality remains unclear, despite prior investigation.^6^, ^11^, ^12^ To address this, it is important that overall diet quality is considered in the context of any associations between UPF and cognition. However, commonly used diet quality indices (e.g., Alternative Healthy Eating Index [AHEI], Dietary Approaches to Stop Hypertension [DASH]) already include UPF‐related items such as soft drinks and processed meats, which may introduce collinearity and obscure the distinct influence of UPF intake. In contrast, adherence to the Mediterranean diet, which is largely based on non‐UPF components, represents a well‐established marker of diet quality consistently associated with cognitive health.^13^, ^14^ The 2024 Lancet Commission on dementia prevention, intervention and care reported that there was insufficient evidence to suggest that reducing UPF consumption would contribute to dementia prevention.^15^ Thus, it is important not only to examine the impact of UPFs on cognition and dementia risk, but also to disentangle the effects of food processing from overall diet quality, which is also vital in elucidating the role of UPF in dementia risk models.

This study examined the cross‐sectional association between UPF intake and cognitive function, as well as dementia risk scores, in Australian adults aged 40 to 70 years enrolled in the Healthy Brain Project (HBP).^16^ This age range captures a critical window for midlife risk accumulation and the emergence of early neurodegenerative changes, both of which may be influenced by diet and lifestyle.^17^, ^18^ Furthermore, this study investigated the extent to which the association between UPF intake and cognitive function and dementia risk scores is explained by overall diet quality.

## 2 METHODS

### 2.1 Study population

The HBP received approval from the Monash University Human Research Ethics Committee (#26855), and all participants provided written informed consent. This study adhered to the STROBE‐nut guidelines for reporting cross‐sectional studies (Supplementary material 1).

The HBP utilized an online platform to recruit dementia‐free adults aged 40–70 years who were living in Australia and fluent in English, as described previously.^16^ Participants were excluded if they self‐reported having a major neurological condition (including dementia, Parkinson's disease, diagnosis of cognitive impairment, multiple sclerosis, traumatic brain injury) or psychiatric disease (including schizophrenia, uncontrolled major depressive disorder), or receiving any approved medication for Alzheimer's disease. Study advertisements targeted individuals with a known or suspected family history of dementia to enrich the sample for genetic risk.^16^

Out of the 6851 participants initially enrolled in the HBP from November 2016 to December 2023 (data freeze 6), 20 were excluded as they were beyond 70 years of age at registration. Of the remaining participants, 2472 had completed assessments of cognition and dietary intake. A total of 2192 participants were included in this cross‐sectional study, after excluding 280 participants due to missing demographic data.

**RESEARCH IN CONTEXT**

1. **Systematic review**: We searched PubMed for studies examining ultra‐processed food (UPF) consumption and cognitive outcomes. While UPFs are linked to over 30 adverse health conditions, emerging evidence suggests potential neurocognitive implications. Several observational studies report associations between higher UPF intake and poorer cognition or accelerated decline, but heterogeneity in study design, UPF definitions, and confounding dietary patterns limits interpretability.
2. **Interpretation**: Our findings reinforce prior evidence that higher UPF consumption is associated with poorer attention and increased modifiable dementia risk. Crucially, these associations persisted after adjusting for overall diet quality, indicating that food processing itself may influence cognitive health beyond nutrient displacement.
3. **Future directions**: Future research should clarify causal mechanisms linking UPFs to cognitive decline, including neuroinflammatory and metabolic pathways. Longitudinal studies with robust dietary assessments and biomarker integration are needed to disentangle UPF effects from broader dietary patterns and inform dementia prevention strategies.

### 2.2 Demographics and health

Demographics (age, sex, race, education, residential address), height, weight, and current smoking status (smoker, non‐smoker, missing) were collected using self‐reported questionnaires. Race was categorized as White and racial minority since several of the other racial categories (African, Asian, Indigenous Australian, Latin American, Other) had very small sample sizes (as low as <0.1%).^19^ Education was categorized as ≤ 12 years (corresponding to high school completion), 13–15 years (corresponding to undergraduate level), and ≥15 years (corresponding to postgraduate level). Residential postcodes were matched with data from the Australian Bureau of Statistics to identify the Index of Relative Socio‐economic Disadvantage (IRSD), since cognitive function and dementia risk have been previously associated with neighborhood‐level socioeconomic status in the HBP population.^19^ IRSD scores were divided into quintiles, with higher values reflecting less disadvantaged groups. Body mass index (BMI) was calculated from weight and height and categorized as underweight/normal weight (≤24.9 kg/m^2^), overweight (25–29.9 kg/m^2^), and obese (≥30 kg/m^2^). Underweight and normal weight categories were combined because only 31 (1.4%) participants had a BMI < 18.5 kg/m^2^. Participants lacking BMI information were categorized as “missing” (*n* = 77). Physical activity information, collected using the International Physical Activity Questionnaire (IPAQ), was categorized as high, moderate, or low (inactive) activity according to the total moderate to vigorous work MET‐minutes per day, as per the continuous scoring guidelines for the IPAQ long format survey.^20^ Participants who did not have information for physical activity were categorized as “missing” (*n* = 219). Cardiometabolic disease history was classified based on self‐reported diagnoses of diabetes, dyslipidemia, or hypertension; participants reporting any of these were considered positive for cardiometabolic disease.

### 2.3 Dietary assessment

Dietary data were collected online on the HBP platform using a validated 130‐item Food Frequency Questionnaire (FFQ) from the European Prospective Investigation into Cancer United Kingdom Norfolk cohort (EPIC‐Norfolk).^21^, ^22^ The FFQ was used to measure habitual food intake over the previous 12 months and took approximately 30 min to complete. Answers range from “never or less than once per month” to “6 + times a day”. Frequencies were converted into grams by multiplying daily equivalent frequencies by sex‐specific portion sizes used in the Australian Cancer Council Victoria's Dietary Questionnaire for Epidemiological Studies.^23^ We derived our own estimates of energy (by food items and total energy intake) and nutrient intakes (e.g., monounsaturated fats and saturated fats) by assigning FFQ food and beverage items to an appropriate energy and nutrient profile based on the 2011–2013 Australian Food and Nutrient (AUSNUT) database.^24^ Total daily grams and energy intakes were estimated. Daily equivalent frequencies, sex‐specific portion sizes and matching of FFQ items to AUSNUT are presented in Supplementary Material 2. All food and beverage items were classified according to the Nova system into one of the four groups: unprocessed/minimally processed foods, processed culinary ingredients, processed foods and UPFs (Supplementary Methods 1). Adherence to the Mediterranean diet was measured using the nine‐item index developed and validated by Trichopolou et al.^25^ (Supplementary Methods 2 and 3).

### 2.4 Cognitive function

All participants completed the Cogstate Brief Battery online on the HBP platform. The validity and acceptability of this battery in unsupervised assessment settings have been demonstrated in previous studies.^26^, ^27^, ^28^ Briefly, participants completed four card‐based cognitive tasks^29^, ^30^: (i) Detection (DET), a measure of processing speed and psychomotor function; (ii) Identification (IDN), a measure of visual attention; (iii) One card learning (OCL), a measure of visual recognition memory set within a continuous recognition paradigm requiring discrimination of novel versus previously presented stimuli; and (iv) One‐back (OBK), a measure of working memory and attention. The primary outcome for DET, IDN, and OBK was reaction time in milliseconds (normalized using a log10 transformation), with lower values indicating faster task completion. The primary outcome for OCL was the proportion of correct responses, normalized using an arcsine square‐root transformation, with higher values indicating greater performance accuracy. All outcomes were standardized into *z*‐scores using the mean and standard deviation of the total sample in the study. To ensure consistency of interpretation, DET, IDN, and OBK outcomes were reverse‐coded so that higher *z*‐scores uniformly reflected better performance. Two composite scores were then computed: (i) an Attention composite, calculated as the average of the standardized scores from DET and IDN; and (ii) a Memory composite, computed as the average of standardized scores from OCL and OBK.

### 2.5 Dementia risk

Dementia risk was estimated using two cardiovascular risk factors, aging, and incidence of dementia (CAIDE) risk scores for a subgroup of participants with the required data available (*n* = 1891). The original CAIDE risk score was developed to predict 20‐year dementia risk among middle‐aged people.^31^ It is based on age, education level, sex, history of hypercholesterolemia and hypertension, physical activity, and BMI, yielding a total score from 0 to 15.^31^ To isolate the part of dementia risk that can plausibly be influenced by diet, we have also used the modified CAIDE dementia risk score previously reported in the HBP,^19^ which considers only modifiable risk factors and is limited to history of hypercholesterolemia and hypertension, physical activity and BMI, ranging from 0 to 7 points. For both CAIDE dementia risk scores, higher scores are indicative of higher dementia risk.

### 2.6 Statistical analysis

Demographic, health and dietary data were described for the overall population and across the population‐stratified quintiles of the dietary contribution of UPF (% grams of total intake). Differences across groups were assessed using linear regression (P‐for‐trend) for continuous variables or Pearson's chi‐squared for categorical variables. The dietary contribution of UPF subgroups (% of total grams/day) was estimated and graphically presented.

The association between UPF intake (as both a continuous variable and categorized into quintiles) and cognitive composite scores was examined using linear regression models adjusted for age, sex, race, education, IRSD, physical activity, smoking status, and cardiometabolic diseases (Model 1). In Model 2, the Mediterranean diet score was added to assess its effect on the association, since diet quality has been associated with both UPF consumption^32^, ^33^ and worse cognitive health.^9^, ^10^ Given that overweight/obesity has been associated with both UPF consumption and worse cognitive function,^1^ in Model 3 we explored its effect on the association by including the covariates in Model 1 with additional adjustment for BMI.

The association between UPF intake (as both a continuous variable and quintiles) and CAIDE dementia risk scores was assessed using linear models adjusted for variables not contained within the risk score; thus, models including the original CAIDE dementia risk score were adjusted for race, IRSD, and smoking status, while models including the modified CAIDE dementia risk score were adjusted for age, sex, race, education, IRSD and smoking status (Model 1). Model 2, for both CAIDE dementia risk scores, was further adjusted for the Mediterranean diet score to examine its effect on the association, since diet quality has been associated with both UPF consumption and dementia risk. Model 3 (further adjusted for BMI) was not performed for these outcomes, as this variable is contained within the risk scores.

Cohen's *f* ^2^ was calculated using UPF intake as a continuous variable to estimate the effect size of UPF on the outcomes. In the models using quintiles of UPF intake, tests of linear trend were carried out by treating quintiles as a single continuous ordinal variable. Collinearity among independent variables was assessed using variance inflation factors (VIF), and was not detected in the regression models.

Sensitivity analyses were performed to test associations with the UPF exposure as percentage of total energy intake, and to test the associations after excluding participants who reported implausible energy intakes (i.e., <500 kcal or >3500 kcal/day for women, and <800 or >4000 kcal/day for men^34^). Participants with implausible energy intake reports were retained in the main analysis to avoid potential selection bias arising from differences in characteristics between plausible and non‐plausible energy reporters.^35^ All statistical analyses were performed with STATA 17 (STATA/SE 17.0 for Windows; StataCorp LLC, US), and a *p* value ≤ 0.05 was considered statistically significant.

## 3 RESULTS

### 3.1 Demographic characteristics

Table 1 summarizes the demographic characteristics of the study sample. Overall, the participants were mostly female, with a mean age of 56.6 years, and younger participants and men reported significantly higher UPF intake. Higher UPF intake was associated with lower educational attainment, obesity and lower adherence to a Mediterranean diet (Table 1).

**TABLE 1:** Characteristics of the study population by quintiles of ultra‐processed food consumption (*n* = 2192).

		Quintiles of UPF (% grams of total intake)**^a^**					
Characteristic	Overall	Q1 (4.1–13.0)	Q2 (13.01–17.2)	Q3 (17.3– 21.7)	Q4 (21.8 –28.2)	Q5 (28.3– 73.4)	*p*‐value
Total participants, *n*	2192	439	438	439	438	438	
Age, mean (SD), years^b^	56.6 (7.1)	57.5 (6.8)	57.2 (6.6)	56.8 (7.0)	56.9 (7.2)	55.0 (7.6)	<0.001
Female, no. (%)^c^	1653 (75.4)	362 (82.5)	359 (82.0)	336 (76.5)	324 (74.0)	272 (62.1)	<0.001
Race, no. (%)^c^							0.899
White or European	1812 (82.7)	363 (82.7)	359 (82.0)	360 (82.0)	369 (84.2)	361 (82.4)	
Racial minority	380 (17.3)	76 (17.3)	79 (18.0)	79 (18.0)	69 (15.8)	77 (17.6)	
Education, mean (SD), years^b^	16.1 (3.4)	16.6 (3.3)	16.1 (3.6)	16.1 (3.3)	15.9 (3.4)	15.9 (3.3)	0.002
Education, no. (%)^c^							0.308
≤12 years	335 (15.3)	57 (13.0)	68 (15.5)	65 (14.8)	72 (16.5)	73 (16.7)	
13–15 years	507 (23.1)	87 (19.8)	113 (25.8)	100 (22.8)	104 (23.7)	103 (23.5)	
≥15 years	1350 (61.6)	295 (67.2)	257 (58.7)	274 (62.4)	262 (59.8)	262 (59.8)	
IRSD, no. (%)^c^							0.120
Q1 (most disadvantaged)	158 (7.2)	25 (5.7)	22 (5.0)	25 (5.7)	39 (8.9)	47 (10.8)	
Q2	291 (13.3)	63 (14.4)	63 (14.4)	51 (11.6)	60 (13.7)	54 (12.3)	
Q3	370 (16.9)	73 (16.6)	69 (15.8)	83 (18.9)	74 (16.9)	71 (16.2)	
Q4	562 (25.6)	112 (25.5)	110 (25.1)	110 (25.1)	109 (24.9)	121 (27.6)	
Q5 (less disadvantaged)	811 (37.0)	166 (37.8)	174 (39.7)	170 (38.7)	156 (35.6)	145 (33.1)	
BMI, no. (%)^c^							<0.001
≤24.99 kg/m	945 (43.1)	226 (51.5)	209 (47.7)	198 (45.1)	165 (37.7)	147 (33.6)	
25 – 29.99 kg/m	711 (32.4)	133 (30.3)	135 (30.8)	141 (32.1)	160 (36.5)	142 (32.4)	
>30 kg/m	459 (20.9)	64 (14.6)	80 (18.3)	89 (20.3)	95 (21.7)	131 (29.9)	
Missing	77 (3.5)	16 (3.6)	14 (3.2)	11 (2.5)	18 (4.1)	18 (4.1)	
Current smoker, no. (%)^c^	69 (3.1)	8 (1.8)	12 (2.7)	17 (3.9)	16 (3.6)	16 (3.6)	0.448
Missing	29 (1.3)	8 (1.8)	3 (0.7)	4 (0.9)	6 (1.4)	8 (1.8)	
Physical activity, no. (%)^c^							0.309
Low	198 (9.0)	30 (6.8)	39 (8.9)	38 (8.7)	42 (9.6)	49 (11.2)	
Moderate	881 (40.2)	160 (36.5)	184 (42.0)	184 (41.9)	184 (42.0)	169 (38.6)	
High	894 (40.8)	199 (45.3)	179 (40.9)	173 (39.4)	172 (39.3)	171 (39.0)	
Missing	219 (10.0)	50 (11.4)	36 (8.2)	44 (10.0)	40 (9.1)	49 (11.2)	
Cardiometabolic diseases, no. (%)^c^	944 (43.1)	189 (43.5)	172 (39.3)	189 (43.0)	203 (46.3)	191 (43.6)	0.528
Missing	13 (0.6)	2 (0.5)	4 (0.9)	4 (0.9)	2 (0.5)	1 (0.2)	
Dietary energy intake, mean (SD), kcal/day^b^	2692 (1003)	2061 (627)	2374 (726)	2662 (831)	2994 (1020)	3372 (1153)	<0.001
Dietary UPF, mean (SD), % g/day^b^	21.1 (9.6)	10.2 (2.0)	15.1 (1.2)	19.5 (1.2)	24.7 (1.9)	36.2 (7.5)	<0.001
Dietary UPF, mean (SD), % kcal/day^b^	40.6 (12.5)	26.6 (7.3)	34.9 (7.7)	40.4 (8.3)	46.3 (8.2)	54.6 (9.0)	<0.001
Mediterranean diet score^b,d^	5.02 (1.63)	5.17 (1.67)	5.15 (1.63)	5.15 (1.63)	4.98 (1.59)	4.67 (1.59)	<0.001

Abbreviations: BMI, body mass index; IRSD, Index of Relative Socio‐economic Disadvantage; SD, standard deviation; UPF, ultra‐processed foods.

^a^ Min–max range of % grams UPF intake per quintile.

^b^ Comparisons across quintiles were performed using linear regression (P‐for‐trend).

^c^ Comparisons across quintiles were performed using Pearson chi‐squared.

^d^ Ranges from 0 (lower adherence to the Mediterranean diet) to 9 (higher adherence representing higher diet quality).

**FIGURE 1:**
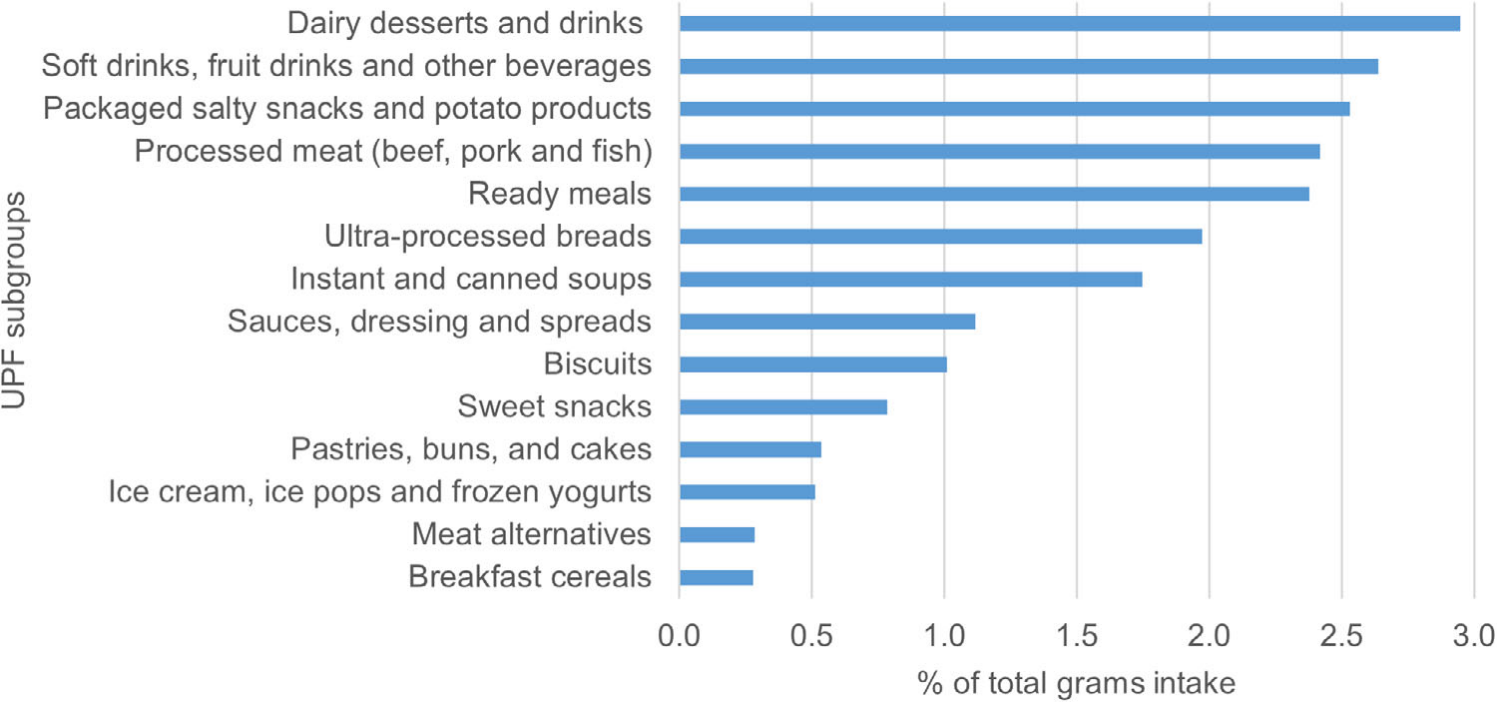
Contribution (%) of UPF subgroups to total grams intake. UPF, ultra‐processed foods.

Overall, UPFs contributed to 21% of total daily weight of all food and beverages consumed and 41% of total energy intake, with a significantly higher proportion of the energy intake in the higher quintiles (Table 1). Across the study population, the most consumed UPFs were dairy‐based desserts and drinks (2.9% of total grams), soft drinks, fruit drinks and other sweetened beverages (2.6%), packaged salty snacks and potato products (2.5%), reconstituted processed meat (2.4%), and ready meals (2.4%) (Figure 1, Supplementary material 3).

### 3.2 UPF intake and cognitive function

**FIGURE 2:**
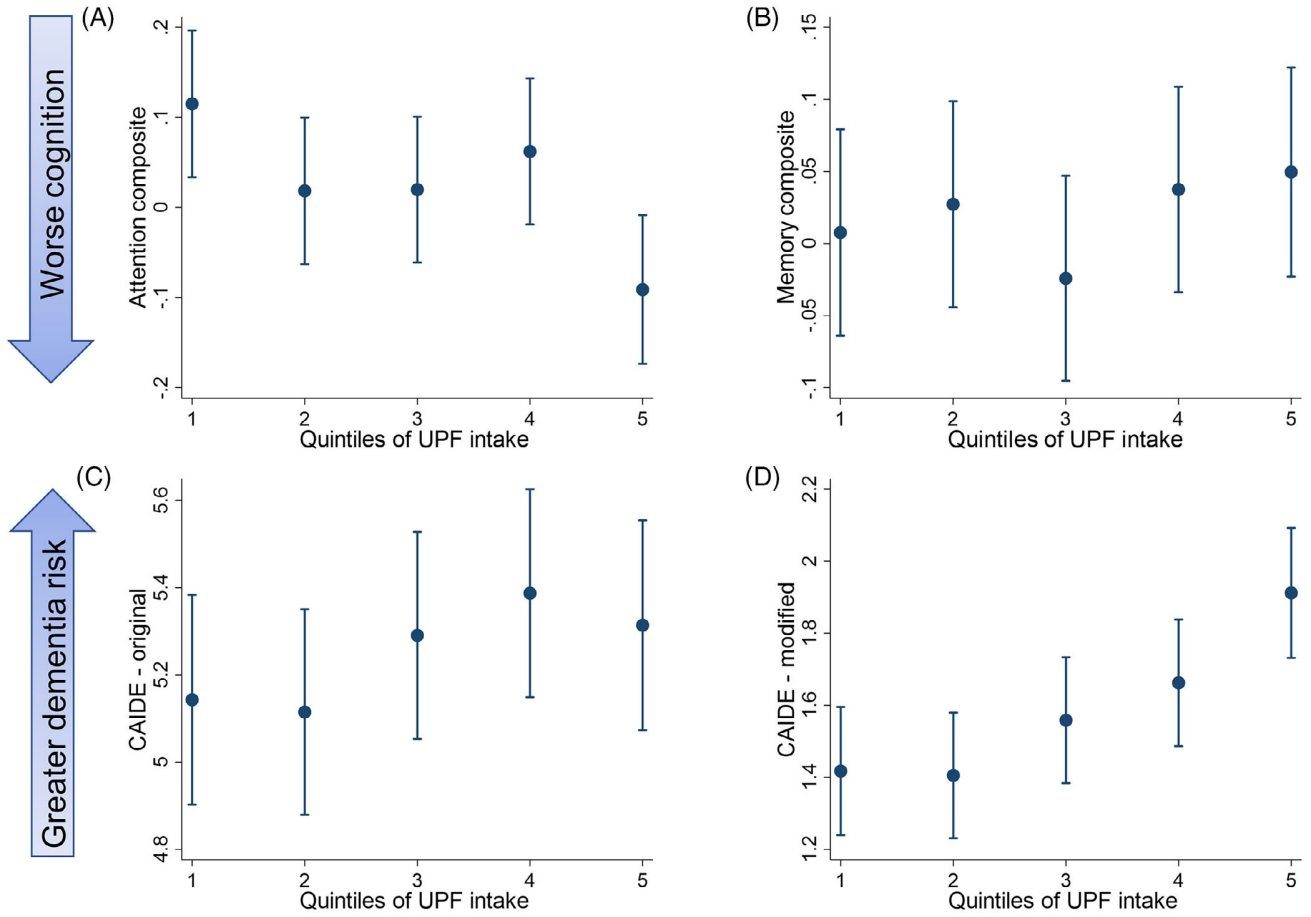
Predicted mean (SD) scores for (A) Attention composite, (B) Memory composite, (C) CAIDE – original, and (D) CAIDE – modified by quintiles of ultra‐processed food consumption. Values are presented as mean and 95% confidence interval. (A, B) Models adjusted for age, sex, race, education, IRSD, physical activity, smoking status and cardiometabolic diseases; C: Model adjusted for race, IRSD, and smoking status; D: Model adjusted for age, sex, race, education, IRSD and smoking status. IRSD, Index of Relative Socio‐economic Disadvantage; SD, standard eviation.

Table 2 summarizes group mean performance on the Cogstate attention and memory composites for each UPF intake quintile. After adjusting for covariates, higher consumption of UPF was associated with poorer attention (Table 3, Figure 2). Model 1 showed that for every 10% increase in UPF consumption, there was a 0.05 decrease in the attention score, and the magnitude of this effect was small (Cohen's *f* ^2^ = 0.03). Similarly, higher quintiles of UPF intake were monotonically associated with lower attention scores, although only Q5 differed significantly from Q1 (Model 1, *P*‐for‐trend = 0.006). No associations were observed between UPF intake and memory (Table 3, Figure 2). The association between UPF intake and cognitive outcomes remained unchanged after further adjusting for Mediterranean diet score and BMI (Models 2 and 3) (Table 3). No differences in these findings were observed in the sensitivity analyses when associations with the UPF exposure as percentage of total energy intake were tested (Supplementary material 4), and when the analysis was limited only to individuals with plausible energy intake (Supplementary material 5).

**TABLE 2:** Cognitive (attention and memory) composite scores, and CAIDE dementia risk scores by quintiles of UPF consumption.

Quintiles of UPF (% grams of total intake)	Attention composite score, *z*‐scores**^a^**	Memory composite score, *z*‐scores**^a^**	Original CAIDE score**^b^**	Modified CAIDE score**^b^**
	Mean (SD)	Mean (SD)	Mean (SD)	Mean (SD)
Q1 (4.1–13.0)	0.08 (0.91)	0.00 (0.81)	5.1 (2.3)	1.4 (1.8)
Q2 (13.01–17.2)	−0.01 (0.94)	0.01 (0.74)	5.1 (2.1)	1.4 (1.7)
Q3 (17.3–21.7)	0.01 (0.87)	−0.02 (0.74)	5.3 (2.3)	1.6 (1.8)
Q4 (21.8–28.2)	0.05 (0.92)	0.03 (0.81)	5.4 (2.5)	1.7 (1.8)
Q5 (28.3–73.4)	−0.02 (0.90)	0.07 (0.75)	5.3 (2.6)	1.9 (1.8)

^a^ *n* = 2192 participants.

^b^ *n* = 1891 participants.

Abbreviations: SD, standard deviation; UPF, ultra‐processed foods.

**TABLE 3:** Association of UPF intake (% grams of total intake) with cognitive (memory and attention) composite scores (*n* = 2192).

				Quintiles of UPF (% grams of total intake)					
	UPF (% grams of total intake)			*β* (95% CI)					
Parameter	*β* (95% CI) **^a^**	*p‐*value	Effect size ^a^, ^b^	Q1	Q2	Q3	Q4	Q5	*p*‐Trend
**Attention composite score**									
Model 1	−0.05 (−0.09, −0.01)	0.012	0.03	–	−0.10 (−0.21, 0.02)	−0.09 (−0.21, 0.02)	−0.05 (−0.17, 0.06)	−0.21 (−0.32, −0.09)	0.006
Model 2	−0.05 (−0.09, −0.01)	0.011	0.03	–	−0.10 (−0.21, 0.02)	0.09 (−0.21, 0.02)	−0.05 (−0.17, 0.06)	−0.21 (−0.33, −0.09)	0.006
Model 3	−0.05 (−0.08, −0.01)	0.012	0.02	–	−0.10 (−0.21, 0.02)	−0.10 (−0.21, 0.02)	−0.05 (−0.16, 0.06)	−0.20 (−0.32, −0.08)	0.009
**Memory composite score**									
Model 1	0.01 (−0.02, 0.04)	0.502	0.002	–	0.02 (−0.08, 0.12)	−0.03 (−0.13, 0.07)	0.03 (−0.07, 0.13)	0.04 (−0.06, 0.14)	0.427
Model 2	0.01 (−0.02, 0.04)	0.497	0.002	–	0.02 (−0.08, 0.12)	−0.03 (−0.13, 0.07)	0.03 (−0.07, 0.13)	0.04 (−0.06, 0.14)	0.424
Model 3	0.01 (−0.02, 0.05)	0.389	0.003	–	0.02 (−0.08, 0.12)	−0.03 (−0.13, 0.07)	0.03 (−0.07, 0.14)	0.05 (−0.05, 0.15)	0.330

*Note*: Model 1: adjusted for age, sex, race, education, IRSD, physical activity, smoking status, and cardiometabolic diseases. Model 2: adjusted for age, sex, race, education, IRSD, physical activity, smoking status, cardiometabolic diseases, and Mediterranean diet score. Model 3: adjusted for age, sex, race, education, IRSD, physical activity, smoking status, cardiometabolic diseases, and BMI.

Abbreviations: BMI, body mass index; CI, confidence interval; UPF, ultra‐processed foods.

^a^ Presented for 10% increase in UPF intake (as % grams of total intake);

^b^ Cohen's f^2^.

^c^ Constant: Attention composite score: Model 1: 1.96 (1.55, 2.37); Model 2: 1.99 (1.57, 2.42); Model 3: 2.01 (1.59, 2.43); Memory composite score: Model 1: 0.52 (0.16, 0.89); Model 2: 0.52 (0.14, 0.89); Model 3: 0.60 (0.23, 0.97).

### 3.3 UPF intake and dementia risk scores

Table 2 summarizes the group mean CAIDE dementia risk score for each UPF intake quintile. When considered continuously, and after adjusting for race, IRSD, and smoking status, greater UPF intake was associated with higher dementia risk (*β* = 0.11; 95% confidence interval [CI] = 0.007, 0.23; Cohen's *f* ^2^ = 0.02; Table 4). However, this association was no longer statistically significant after adjustment for the Mediterranean diet score. Higher UPF intake was also associated with greater dementia risk, as measured using the modified CAIDE dementia risk score, and the strength of this association was moderate in models both with and without adjustment for the Mediterranean diet (*β* = 0.24; 95% CI = 0.16, 0.32; Cohen's *f* ^2^ = 0.16–0.17; Table 4). The findings remained unchanged when considering UPF exposure as percentage of total energy intake (Supplementary material 6), and when the analysis was limited to individuals with plausible energy intake (Supplementary material 7).

**TABLE 4:** Association of UPF intake (% grams of total intake) with CAIDE dementia risk scores (*n* = 1891).

				Quintiles of UPF (% grams of total intake)					
	UPF (% grams of total intake)			β (95% CI)**^e^**					
	β (95% CI)**^a^**	*p‐*Value	Effect size**^a^** ^,^ **^b^**	Q1	Q2	Q3	Q4	Q5	*p*‐Trend
**CAIDE score** ^c^									
Model 1	0.11 (0.007, 0.23)	0.037	0.02	–	−0.03 (−0.36, 0.31)	0.15 (−0.19, 0.48)	0.24 (−0.09, 0.58)	0.17 (−0.17, 0.51)	0.110
Model 2	0.10 (0.00, 0.22)	0.061	0.03	–	−0.03 (−0.37, 0.30)	0.14 (−0.20, 0.48)	0.23 (−0.11, 0.57)	0.14 (−0.20, 0.48)	0.155
**Modified CAIDE score** ^d^									
Model 1	0.24 (0.16, 0.32)	<0.001	0.17	–	−0.01 (−0.26, 0.24)	0.14 (−0.11, 0.39)	0.24 (−0.005, 0.49)	0.49 (0.24, 0.75)	<0.001
Model 2	0.24 (0.15, 0.32)	<0.001	0.16	–	−0.01 (−0.26, 0.23)	0.14 (−0.11, 0.39)	0.24 (−0.01, 0.49)	0.48 (0.23, 0.74)	<0.001

^a^ Presented for 10% increase in UPF intake (as % grams of total intake).

^b^ Cohen's f^2^.

^c^ Model 1: adjusted for race, IRSD, and smoking status; Model 2: adjusted for race, IRSD, smoking status, and Mediterranean diet score.

^d^ Model 1: adjusted for age, sex, race, education, IRSD, and smoking status; Model 2: adjusted for adjusted for age, sex, race, education, IRSD, smoking status, and Mediterranean diet.

^e^ Constant: CAIDE score: Model 1: 5.37 (4.62, 6.13); Model 2: 5.64 (4.81, 6.48); Modified CAIDE score: Model 1: ‐1.20 (‐2.15, −0.25); Model 2: ‐1.09 (‐2.07, −0.11).

Abbreviations: CI, confidence interval; IRSD, Index of Relative Socio‐economic Disadvantage; UPF, ultra‐processed foods.

## 4 DISCUSSION

In this cross‐sectional study of middle‐aged and older Australian adults, higher UPF consumption was associated with poorer attention and elevated modifiable dementia risk, supporting previous evidence.^6^, ^9^, ^10^ Further, these associations were independent of adherence to the Mediterranean diet, a well‐established marker of diet quality associated with cognitive health,^13^, ^14^ suggesting the link between diet and cognitive function extends beyond diet quality and displacement of whole foods alone, to include mechanisms linked to the degree of food processing.^36^

In this study, high UPF intake was not clearly associated with the original CAIDE dementia risk score, but was moderately associated with the modified CAIDE score, which reflects only modifiable cardiometabolic risk factors. Although the modified CAIDE score has not been validated as a dementia risk tool, it offers exploratory insight into how UPFs may relate to modifiable risk pathways relevant to late‑life cognitive health. Each 10% increase in UPF consumption was associated with a 0.05‐point reduction in attention scores, with the strongest association observed among individuals in the highest quintile of UPF intake (≥28.3% of total grams consumed). In our results, only the fifth quintile of UPF intake showed a statistically significant difference from the first quintile for attention scores. This likely reflects the broader interval of UPF intake in Q5 compared with the narrower ranges in Q1–Q4. Importantly, this does not undermine the significant linear trend observed across quintiles. Trend analyses assess the overall gradient of association across exposure distribution, rather than isolated contrasts between categories, and continuous models generally offer greater statistical power to detect subtle relationships. Our findings align with previous research, which has also shown that higher UPF intake is associated with poorer cognitive outcomes,^9^, ^10^ particularly attention and executive function.^6^, ^7^ The observation in this study that UPF intake was not associated with memory outcomes has also been observed previously.^6^ Given that attention is foundational to many cognitive operations, such as learning, problem‐solving, and memory formation, it is plausible that early disruptions in attention may precede broader cognitive impairments.^37^, ^38^ However, the evidence base remains inconclusive,^39^ and further research is needed to clarify the temporal dynamics of these associations. Given that a 10% increase in UPF intake corresponds to approximately 150 g/day (equivalent to a standard packet of potato chips) based on the average daily per capita food intake of 1.5 kg in the Australian population,^40^ these results suggest that even small daily increases in UPF consumption may have measurable cognitive consequences with an impact on dementia risk.

Food ultra‐processing often disrupts the food matrix, reduces whole‐food constituents (e.g., phytochemicals, vitamins, and minerals), and introduces potentially harmful substances such as bisphenols, phthalates, or processing‐derived compounds like acrylamide, which may contribute to adverse neurocognitive outcomes.^41^, ^42^ UPFs are also associated with increased risk for chronic diseases such as diabetes, hypertension, obesity, and high LDL cholesterol, which are major contributors to dementia risk that collectively account for approximately 12% of all dementia cases globally.^15^ These cardiometabolic pathways may help explain the observed negative association between UPF intake and attention, but not memory, given the higher sensitivity of attention and executive function to environmental and physiological stressors that can disrupt cerebrovascular integrity.^43^ UPFs may contribute to cerebrovascular lesions, which in turn impair cognitive domains reliant on vascular health.^43^ Supporting this hypothesis, neuroimaging studies have shown that diet can significantly influence brain structures: adherence to healthy dietary patterns is associated with greater white matter volume,^44^ whereas Western‐style diets are linked to reduced hippocampal volume.^45^ Further, UPFs may have adverse effects on brain health through the microbiota–gut–brain axis. Evidence from animal studies suggests that ingredients commonly found in UPFs, such as emulsifiers, food preservatives and colourants, as well as compounds resultant from food processing (e.g. advanced lipoxidation and glycation end‐products and acrylamide), disrupt the gut microbiota, leading to a reduction in short‐chain fatty acids production, increased intestinal permeability, and persistent inflammation.^46^, ^47^, ^48^ These alterations in the gut environment may impair neurotransmission, cause microglia activation to trigger neuroinflammation, and cause neuronal death.^49^ It is possible that one or more of these links between UPF and brain health could be underlying the current findings; however, future research is required to further elucidate this.

In this study, we used the Nova system, which classifies foods according to the extent and purpose of industrial processing rather than their nutrient composition. This processing‑focused approach means that some foods labelled as ultra‑processed may still be considered health‑promoting within certain dietary guidelines or diet‑quality indices. We acknowledge that Nova simplifies complex food, but it provides a complementary perspective on dietary patterns and is not intended to substitute nutrient‑based analyses.^50^ Because our research question centered on the role of food processing itself, we considered Nova the most appropriate framework and therefore did not further disaggregate UPFs into individual food categories.

The population assessed in this study is considerably unique as the majority of participants reported a first‐ or second‐degree family history of dementia, suggesting they are at higher risk of cognitive decline while remaining free of cognitive impairment or dementia. Assessing dementia risk in midlife using CAIDE scores is particularly relevant, as this life stage offers a key opportunity to address modifiable risk factors before neuropathological changes develop, ultimately reducing the long‑term burden of dementia on individuals, families, and health systems.^15^ Another strength of our study is the identification of UPFs in the FFQ by an international team of nutrition experts in this topic^25^ and the measurement of usual intake using a comprehensive FFQ. However, the study limitations must be acknowledged. While the HBP's online platform offers wide‐reaching recruitment to access geographically varied samples, it limits the ability to verify self‐reported data. The EPIC‐Norfolk FFQ has not been validated for the Australian population, and the use of a self‐reported FFQ is subject to recall bias and measurement error. Nevertheless, our sensitivity analysis revealed that misreporting had no impact on the results. The FFQ was not specifically developed to measure UPFs or to reflect the Australian context; therefore, some misclassification of items may have occurred despite several strategies applied to minimize error.^51^ Although the FFQ items may not capture the complexity of dietary patterns, previous analysis of EPIC data (which derived the FFQ) shows that the Nova system accurately captured UPF consumption, as reflected by stronger correlations with biomarkers.^51^ Additionally, the sample was mostly comprised of women and people with higher education and socio‐economic status compared to the broader Australian population; therefore, the generalizability of the findings is limited. However, the dietary contribution of UPFs in this study (40.6% of total energy intake) is comparable to levels reported for the broader Australian population in a similar age group,^52^ suggesting that the direction of the observed associations may not differ substantially at the population level. Finally, even though the cross‐sectional design of this study limits causal inference by not establishing temporal relationships and may be subject to residual confounding, we acknowledge the importance of the findings in a population of middle‐aged adults, which contribute to the existing body of research on the cognitive risks associated with UPF consumption. Future research should prioritize longitudinal and interventional studies that can establish temporal and causal relationships. Integrating biomarkers, neuroimaging, and microbiome data will be essential to clarify these pathways and inform targeted dietary guidelines for dementia prevention.

## 5 CONCLUSIONS

Our findings suggest that UPF consumption is associated with higher modifiable dementia risk and has detrimental effects on cognitive function, particularly on attention performance, independent of overall diet quality; however, no association was observed between UPF and memory. By identifying food processing as a distinct contributor to poorer cognitive outcomes, our study supports the need to refine dietary guidelines for optimal cognitive function, accounting not only for nutrient composition but also for the degree of processing.

## CONFLICT OF INTEREST STATEMENT

The authors declare no conflict of interest. Author disclosures are available in the Supporting information.

## CONSENT STATEMENT

The HBP received approval from the Monash University Human Research Ethics Committee (#26855), and all participants provided written informed consent.

## Supporting information



Supporting Information

Supporting Information

Supporting Information

Supporting Information
