# Supplementary material for: Ultra‐processed food intake, cognitive function, and dementia risk: A cross‐sectional study of middle‐aged and older Australian adults
Source: Alzheimers Dement (Amst). 2026 Apr 23;18(2):e70335. doi: 10.1002/dad2.70335 (PMC13104065; doi:10.1002/dad2.70335)
Supplement: Supplementary file 1 — Supporting Information [file DAD2-18-e70335-s001.docx]

**Ultra-processed food intake and cognitive function and dementia risk: a cross-sectional study of middle-aged and older Australian adults**

**Supplementary methods**

**Supplementary methods 1 -** Food classification according to Nova

All food and beverage items were classified according to the Nova system, which considers the extent and purpose of industrial food processing, into one of the four mutually exclusive groups: unprocessed or minimally processed foods, processed culinary ingredients, processed foods, and UPFs [1]. We followed the classification methodology previously applied in the EPIC-Norfolk [2]. UPFs included items such as mass-produced packaged breads, crisps, soft drinks, sausages and other reconstituted meats, sauces and spreads. Details of the identification of UPFs in the EPIC FFQ have been extensively described elsewhere [2]. The primary exposure variable was UPF intake, expressed as the percentage of total food weight (grams) and analysed both continuously and by quintiles. This weight-based metric was chosen to account for non-caloric items (e.g., artificially sweetened beverages). Additionally, the proportion of total energy intake derived from UPFs was estimated.

**Supplementary methods 2 –** Mediterranean diet score

Adherence to the Mediterranean diet was measured using the 9-item index developed and validated by Trichopolou et al. [3]. Food and nutrient intakes were scored based on the following components: (i) vegetables, (ii) legumes, (iii) fruits and nuts, (iv) cereals, (v) fish and seafood, (vi) the ratio of monounsaturated fats to saturated fats (MUFA:SFA), (vii) dairy products, (viii) meat and meat products, and (ix) alcohol. Daily gram intake derived from the FFQ was used to estimate sex-specific median intakes and served as cut-off points for scoring. Participants received a score of 1 if their intake was above the median for vegetables, legumes, fruits and nuts, cereals, fish and seafood, and the MUFA:SFA ratio. Conversely, a score of 1 was assigned for intake levels below the median for dairy products, meat, and meat products. The MUFA:SFA ratio was derived using the AUSNUT 2011-13, as described previously. Reported alcohol consumption was converted into daily equivalent intake, with alcohol content quantified using Australian standard drink guidelines [4]. Participants consuming alcohol in moderation (up to 20 g/day, equivalent to two standard drinks) received a score of 1, while those consuming either no alcohol (0 g/day) or excessive amounts (>20 g/day) received a score of 0. The total Mediterranean diet score ranged from 0 to 9, with higher scores indicating greater adherence to the Mediterranean dietary pattern. Variables used to create the score are presented in Supplementary Material 3.

**Supplementary methods 3 –** Components and scoring methods of the Mediterranean Diet Score.

| **Dietary indicator** | **HBP variables** | **Indicator foods^a^** | **Criteria for scoring** |
| --- | --- | --- | --- |
| 1. Vegetables (excluding potatoes, legumes or fruit juice) | veg_beetroot  veg_broccoli  veg_sprouts  veg_cabbage  veg_carrots  veg_cauliflower  veg_coleslaw  veg_garlic  veg_beans  veg_green_salad  veg_leeks  veg_marrow  veg_mushrooms  veg_onions  veg_parsnips  veg_spinach  veg_peppers  veg_sweetcorn  veg_tomatoes  soups_veg_soup  veg_cress  veg_beansprouts  veg_peas  veg_avocado | Mixed vegetable, vegetable pieces, avocado, beetroot, broccoli, butternut squash, cabbage/kale, carrot, cauliflower, celery, courgette, cucumber, garlic, leek, lettuce, mushroom, onion, olives, parsnip, pea, side salad, sweet pepper, spinach Sprouts, sweetcorn, fresh tomato, tinned tomato, green bean, turnip/swede, watercress, other vegetables, homemade soup (vegetables) | Sex-specific median intakes used as cut points. Intakes (for indictors 1-6) above median score 1 and intakes below the median score 0. |
| 1. Legumes | veg_baked_beans  veg_lentils | Baked bean, pulses, broad bean, homemade soup (pulses) |  |
| 1. Fruits and nuts | fruit_apples  fruit_bananas  fruit_dried  fruit_grapefruit  fruit_grapes  fruit_melon  fruit_peaches  fruit_strawbs  fruit_oranges  soups_peanut  sweets_nuts  fruit_pears  drinks_fruit_juice | Stewed fruit, prune, dried fruit, mixed fruit, apple, banana, berry, cherry, grapefruit, grape, mango, melon, orange, satsuma, peach/nectarine, pear intake, pineapple, plum, other fruit Orange juice, grapefruit juice, pure fruit/vegetable juice Unsalted peanuts, unsalted nuts, types of spreads/sauces consumed (Peanut butter), seeds |  |
| 1. Cereals | cereals_cereal  bread_brown potatoes_rice_brown  cereals_porridge bread_white potatoes_pasta_wg potatoes_rice_white  bread_wholemeal  potatoes_pasta_wholemeal | Porridge, muesli, oat crunch, plain cereal, bran cereal, whole-wheat cereal, other cereal Bread consumed, sliced bread (mixed; wholemeal; seeded; other), baguette (mixed; wholemeal; seeded; other), bap (mixed; wholemeal; seeded; other), bread roll (mixed; wholemeal; seeded; other), other bread White pasta, wholemeal pasta, white rice, brown rice, couscous, other grain Homemade soup, ingredients in homemade soup (pasta) |  |
| 1. Fish and seafood | meat_fish_roe  meat_fish_oily meat_fish_white meat_shellfish | Tinned tuna, oily fish, white fish, prawns, lobster/crab, shellfish, other fish Homemade soup, ingredients in homemade soup (fish) |  |
| 1. Monounsaturated/saturated fats ratio |  | Monounsaturated fats, saturated fats |  |
| 1. Dairy products | dairy_cheese_med drinks_cocoa  dairy_cheese_lowfat dairy_full_fat_yog dairy_low_fat_yog  milk | Milk, milk added to cereal Low fat hard cheese, low fat cheese spread, cottage cheese Yogurt (low fat yogurt consumer; full fat yogurt consumer) Goat's cheese, hard cheese, soft cheese, blue cheese, cheese spread, feta, mozzarella, other cheese Dairy smoothie, latte, added milk to instant coffee, added milk to filtered coffee, added milk to espresso, added milk to other coffee type, added milk to standard tea, added milk to rooibos tea, cappuccino | Sex-specific median intakes used as cut points. Intakes (for indictors 7-8) below median score 1 and intakes below the median score 0. |
| 1. Meat and meat products | meat_bacon  meat_beefs  meat_burgers  meat_spam  dairy_eggs  meat_ham  meat_lamb  meat_liver  soups_meat_soup  meat_pork  meat_sausages | Beef, pork, lamb, other meat Whole egg, omelette, eggs in sandwiches, scotch egg, other egg Homemade soup, ingredients in homemade soup (meat), sausage, bacon, ham |  |
| 1. Alcohol* | drinks_wine 13.5%  drinks_beer 3.5%  drinks_port 17.5%  drinks_spirits 40% | Red wine, rose wine, white wine Beer/cider Fortified wine, spirits intake, other alcohol | No more than 2 drinks/day = 1; Never drink or over 2 drinks/day = 0. |

*The following conversions were applied: Wine (100 mL, 13.5%) = 1 standard drink (10 g alcohol); Beer (285 mL, 3.5%) = 0.8 standard drinks; Port (60 mL, 17.5%) = 0.9 standard drinks; Spirits (30 mL, 40%) = 1 standard drink. The total alcohol content consumed per day was then calculated.

**Supplementary methods - References**

1. Monteiro CA, Cannon G, Levy RB, Moubarac JC, Louzada ML, Rauber F, Khandpur N, Cediel G, Neri D, Martinez-Steele E *et al*: **Ultra-processed foods: what they are and how to identify them**. *Public Health Nutr* 2019, **22**(5):936–941.

2. Huybrechts I, Rauber F, Nicolas G, Casagrande C, Kliemann N, Wedekind R, Biessy C, Scalbert A, Touvier M, Aleksandrova K *et al*: **Characterization of the degree of food processing in the European Prospective Investigation into Cancer and Nutrition: Application of the Nova classification and validation using selected biomarkers of food processing**. *Front Nutr* 2022, **9**:1035580.

3. Trichopoulou A, Orfanos P, Norat T, Bueno-de-Mesquita B, Ocké MC, Peeters PH, van der Schouw YT, Boeing H, Hoffmann K, Boffetta P *et al*: **Modified Mediterranean diet and survival: EPIC-elderly prospective cohort study**. *BMJ* 2005, **330**(7498):991.

4. National Health and Medical Research Council: **Australian Guidelines to Reduce Health Risks from Drinking Alcohol**. In*.* Commonwealth of Australia, Canberra 2020.
